# Designing Hybrids Targeting the Cholinergic System by Modulating the Muscarinic and Nicotinic Receptors: A Concept to Treat Alzheimer’s Disease

**DOI:** 10.3390/molecules23123230

**Published:** 2018-12-07

**Authors:** Daniela Volpato, Ulrike Holzgrabe

**Affiliations:** Department of Pharmaceutical and Medicinal Chemistry, Institute of Pharmacy, University of Würzburg, Am Hubland, 97074 Würzburg, Germany; daniela.volpato@uni-wuerzburg.de

**Keywords:** AChE inhibitor, Alzheimer’s disease, bitopic ligand, cholinergic system, hybrid molecules, muscarinic receptors, nicotinic receptors

## Abstract

The cholinergic hypothesis has been reported first being the cause of memory dysfunction in the Alzheimer’s disease. Researchers around the globe have focused their attention on understanding the mechanisms of how this complicated system contributes to processes such as learning, memory, disorientation, linguistic problems, and behavioral issues in the indicated chronic neurodegenerative disease. The present review reports recent updates in hybrid molecule design as a strategy for selectively addressing multiple target proteins involved in Alzheimer’s disease (AD) and the study of their therapeutic relevance. The rationale and the design of the bifunctional compounds will be discussed in order to understand their potential as tools to investigate the role of the cholinergic system in AD.

## 1. Introduction

Alzheimer’s disease (AD) is the most common form of dementia among geriatric people since the beginning of the 21st century. In 2016, over 47.5 million people around the world suffered from dementia. The World Health Organization hypothesizes that the number may rise to 75.6 million by 2030. The clinical condition of AD is characterized by a progressive loss of memory and other intellectual abilities up to serious levels where the daily life is severely affected [1]. Of note, no definitive cause and no known cure have been found yet.

The characteristic features of the neurodegenerative decline include a decrease in levels of acetylcholine (ACh) followed by dysfunction and eventual death of cholinergic neurons. Furthermore, soluble β-amyloid oligomers (sAβ), β-amyloid fibrils (fAβ) misfolding and aggregation as well as formation of tau protein tangles in the nervous tissue of brain occur as a result of tau-protein hyperphosphorylation [1,2,3]. Water-soluble Aβ aggregates, detected in human brain lysates from AD patients, were found to be more toxic than their insoluble fibrillar counterparts by Li et al. [4]. Several other co-factors were hypothesized, such as oxidative stress and heavy metal dyshomeostasis [5].

Etiologic evidence exists between AD, cholinergic, and glutamatergic neurochemical systems. ACh is a crucial neurotransmitter responsible for mental and learning abilities. In AD patients, a dramatic decrease in cholinergic innervation in the cortex and the hippocampus is related to the loss of neurons in the basal forebrain [6]. Presynaptic cholinergic dysfunctions, loss of the cholinergic neural network, and overactivation of acetylcholinesterase (AChE), which leads to weakened neurotransmission, support the cholinergic hypothesis of the disease. This assumption suggests that cholinergic restoration therapy could be useful for alleviating the cognitive symptoms [7]. Another neurochemical hypothesis is mediated by *N*-methyl-d-aspartate (NMDA). Glutamate is an excitatory neurotransmitter for NMDA receptors which are essential in cognitive processes. However, in addition to the physiological stimulation, glutamate receptors cause neuronal damage due to excitotoxicity under certain circumstances [8].

**Figure 1:**
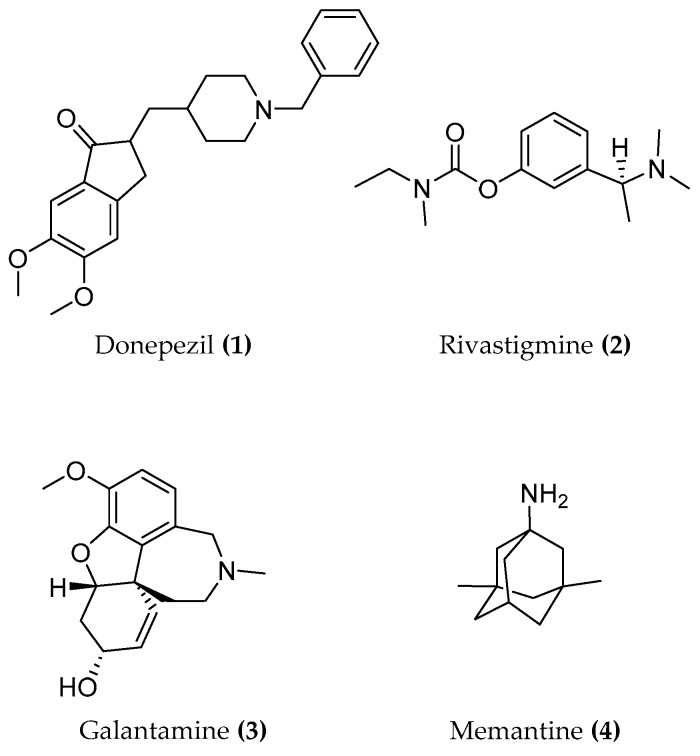
Structures of donepezil, rivastigmine, galantamine and memantine.

The conventional pharmacotherapy of AD makes use of compounds which inhibit the enzyme AChE (donepezil, rivastigmine, and galantamine) in order to increase the levels of acetylcholine in the nervous tissue of the brain ((1–3), Figure 1). However, these compounds can improve the cognitive deficits of the disease only for a couple of months before losing activity. The shortness of the effect could be due to a decrease of the AChE itself in the relevant regions of the brain [9]. Later in time, another drug was introduced for the treatment of AD, i.e., memantine ((4), Figure 1) being a glutamatergic antagonist which protects nerve tissue against glutamate-mediated excitotoxicity. Unfortunately, both classes of drugs only provide a symptomatic relief without preventing the progression of the disease, a fact which is not surprising given the intricacy of the pathology. Thus, there is a desperate need to find selective and powerful drugs to improve the treatment of AD [1].

So far, developing new leading compounds with a lower risk-to-benefit ratio where benefits clearly overcome the side effects has not been accomplished yet due to the complexity and incomplete understanding of the pathophysiological processes in AD. Progress through clinical trials is limited by poor selectivity of candidate molecules and a lack of knowledge of the exact role of proteins considered as crucial targets. To combat this complex multifactorial disease, the challenge is to discover which and how many biological targets have to be modulated by developing powerful and selective drugs. Hence, the idea of creating hybrid molecules containing two discrete recognition units linked through a spacer, resulting in the so-called hybridization approach, is followed. A supplemental molecular segment next to the first standard pharmacophore could serve to address different molecular targets or distinct domains of the same protein. In addition to traditional methods, various modern drug discovery approaches must be investigated in parallel and complementarily to bring new hopes in this therapeutic field. The resulting hybrid molecules can serve as tools for opening new interesting perspectives in medicinal chemistry with more promising therapeutic opportunities to fight AD (for discussions on the further potential of the multifunctional approach see ref. [10]). 

## 2. The Cholinergic Hypothesis of Memory Dysfunction

**Figure 2:**
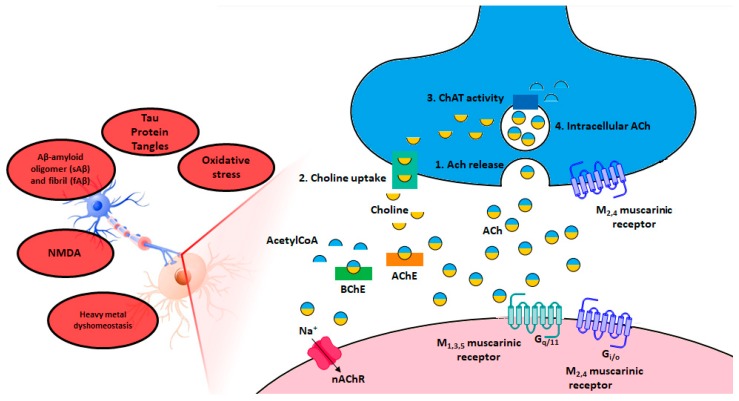
In the red circles some hallmarks of Alzheimer’s disease are reported, the cholinergic hypothesis is emphasized through the schematic representation of a pre and postsynaptic neuron. Zoom in on the cholinergic synapse in particular biosynthesis, storage, release, hydrolysis of ACh and AChR locations are illustrated (modified from Auld et al. [24]).

The substantial loss of presynaptic cholinergic neurons in human postmortem Alzheimer’s-diseased brains, the dysfunction of cholinergic markers such as ACh, choline, and choline acetyl transferase (ChAT, an enzyme responsible for ACh production) as well as the consequent loss of cognitive skills and the mental improvement observed in patients upon artificial restoration of cholinergic activity unequivocally confirm the validity of the cholinergic hypothesis in AD [2]. Acetylcholine receptors (AChRs) propagate the cognitive ability and consist of two primary members namely muscarinic (mAChRs) and nicotinic receptors (nAChRs). While mAChRs are G-protein coupled receptors (GPCRs), nAChRs are ligand gated ion channels. After release, ACh is hydrolyzed to choline and acetate in the synaptic cleft by two cholinesterases, i.e., the AChE and the butyrylcholinesterase (BChE) (see Figure 2). Over the course of the disease, the concentration of AChE decreases by 85%, whereas high levels of BChE were found to influence the aggregation of Aβ, the main component of the amyloid plaques found in the brains of Alzheimer patients [11,12]. Furthermore, synaptic dysfunction and neuronal cell death in vitro [13,14,15,16] as well as impaired behavioral performance in animal models [17,18,19] seem to be directly correlated with sAβ aggregates [20]. For this reason, mAChRs, nAChRs, as well as the hydrolases AChE and BChE are important targets in the treatment of AD [21,22,23]. 

## 3. Hybrid Compounds as Drug Discovery Tools in AD

Due to the absolute relevance of the cholinergic system in AD it may be desirable to address the involved cholinergic receptors and enzymes simultaneously by means of a multi-target strategy and/or selectively through a dualsteric approach. This can be realized by addressing different binding pockets of the same protein’s surface with one hybrid molecule or different proteins involved in the same pathology. “Bivalent ligand” and “hybrid” are the general terms defining compounds which are composed of two functional pharmacophores linked by a spacer [25].

### 3.1. One Single Molecule—Different Targets

Because of the multifactorial dimension of AD and the difficulty to apply the classical “one target—one disease” concept [26], the multitarget compounds approach has often been considered in recent years [27]. In this review, only hybrids intended to activate the cholinergic system will be described. The advantages of the multipotent strategy are obvious, but it remains absolutely challenging. In fact, molecular hybridization involves integration of the structure-activity relationships of two individual chemical species into a single molecular entity which should result in a balance of potency, efficacy, and selectivity for all the targets. The new hybrids can be designed with different degrees of pharmacophore overlapping, with linking or merging techniques, and using distinct connection positions of the active moieties. Furthermore, the combination of several pharmacophoric units usually results in large molecules which have limits in terms of molecular weight, lipophilicity, permeability of membranes, and hydrogen bonds acceptance and donation [27]. The technique lends itself well to address the cholinergic system in AD by concomitant inhibition of AChE and direct muscarinic and nicotinic activation.

### 3.2. One Single Molecule—Different Binding Pockets of a Protein’s Surface

As already mentioned, the hybridization strategy can be used to obtain molecules containing two pharmacophoric entities able to bind topographically different binding sites of the same target protein, e.g., the endogenous orthosteric site and the allosteric one. Allosterism, first defined to describe the modification of any enzyme activity by the binding of ligands to sites that were different from the substrate-binding site itself [28], is widely explored for GPCRs, representing the largest class of drug targets in the human genome [25]. It provides the great opportunity of selectively affecting one GPCR subtype and simultaneously avoiding the problem of targeting the structurally conserved orthosteric binding site [29]. Novel drugs targeting GPCRs do not enter the market as frequent as expected due to unwanted off-target effects resulting from the lack of selectivity within a distinct receptor family [30].

#### 3.2.1. Allosteric Modulation

**Figure 3:**
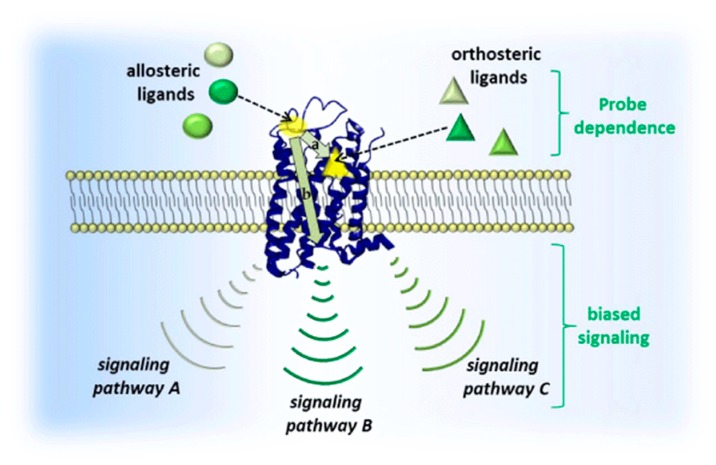
Schematic of GPCR signal transduction: allosteric modulators (spheres) bind to a topographically different site (yellow sphere) than the orthosteric site (yellow triangle) which hosts an orthosteric ligand (triangles). Reproduced with permission from [35].

GPCRs constitute a superfamily of integral membrane proteins characterized by seven transmembrane domains being connected by three extracellular (EL) and intracellular (IL) loops [31]. Allosteric modulators do not bind to the orthosteric binding site but act at a distinct binding site (i.e., an allosteric site) to either potentiate or inhibit activation of the receptor by its endogenous ligand [32]. An innovative perspective in drug development takes advantage of allosteric binding sites which usually altered between the different receptor subtype throughout the evolutionary processes. Conformational changes of the receptor upon allosteric ligand binding can modify the activity of an orthosteric agent in terms of the binding affinity and/or the downstream efficacy. This means emphasizing signals to some pathways over others among the various possible combinations which are associated with receptor activation, a phenomenon known as bias signaling (cf. Figure 3) [33,34].

Besides subtype selectivity, allosteric modulators offer the advantage of saturable effects. Since most of them lack agonistic activity, they can perfectly “fine-tune” any orthosteric ligand activity to a ceiling level above which no further modulation occurs; this means they can be administered with higher doses and lower propensity to toxicity compared to classic agonists or antagonists [25]. 

Since the discovery of allosterism, several theories were postulated in order to identify and quantify this phenomenon: Ehlert’s ternary complex model is one of the most significant methods describing how two distinct ligands, orthosteric and allosteric, may influence the binding and functioning of each other [36]. The cooperativity level, indicated by the cooperativity factor α, distinguishes between positive and negative allosteric modulators (PAMs and NAMs) and neutral allosteric ligands (NALs). It is important to keep in mind that the obtained cooperativity is always referred to a single allosteric modulator for a specific orthosteric probe and that an accurate interpretation of a combination of functional and binding assays is necessary to understand the actual kind of modulation. Probe dependency and cooperativity reveal a new level of subtype selectivity rather than affinity, a phenomenon which was termed “absolute subtype selectivity”. For example, the binding of ACh is selectively enhanced at the M4 muscarinic receptor by thiochrome binding to the allosteric site, even though this allosteric modulator shows almost equal affinity for all muscarinic subtypes [37].

A particular beneficial therapeutic meaning may be associated with allosteric agonist compounds which differ from pure allosteric modulators. They are able to directly activate a receptor by binding to an allosteric binding site even in the absence of an orthosteric ligand. These allosteric agonists might be more suitable for the treatment of AD, either because of the reduced endogenous tone of neurotransmitters or even their complete lack [38]. 

#### 3.2.2. Bitopic Ligand Principle

The structural diversity among the extracellular vestibules allowed an innovative medicinal chemistry strategy for the design of novel ligands, i.e., hybrid compounds. They are characterized by chemical fragments which allow an interaction with both orthosteric and allosteric recognition sites. 

Merging the best of both allosteric and orthosteric worlds in one ligand launches the bitopic or dualsteric compounds [39,40], a strategy derived from the “message-address” concept by Schwyzer [41] which was first applied to GPCRs by Portoghese et al. [42]. This concept explicitly underlines the orthosteric/allosteric combination, in opposite to the more general umbrella term bivalent [25]. The orthosteric interaction provides high affinity binding and activation/inhibition of a receptor (message), whereas the allosteric interaction yields receptor subtype-selectivity (address) and may modulate both efficacy and intracellular signaling pathway activation [43].

**Figure 4:**
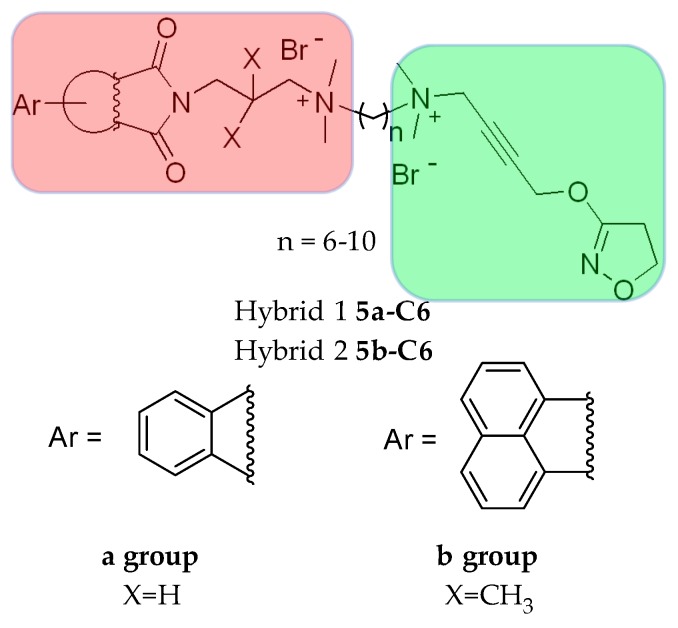
Structures of bitopic hybrids. **5a-C6, 5b-C6** M2 muscarinic hybrid. Green: orthosteric building block, red: allosteric building block.

In fact, the transduction pathway of GPCRs include a large set of intracellular signaling proteins like different G proteins, GRKs (protein coupled receptor kinase), and arrestins [44]. Such hybrid molecules would allow to selectively identify therapeutically relevant signaling pathways, attenuate harmful signals, or selectively block the ability of the agonist to produce unselective signals resulting in side effects [45]. The recent functional screening of biased ligands using multiple assay formats suggest that the phenomenon represents an existing and relevant therapeutic opportunity for the treatment of different disorders [43]. The dualsteric principle was validated through numerous bitopic ligands examples: 

Iper-6-phth ((**5a-C6**), Figure 4) and iper-6-naph ((**5b-C6**), Figure 4) are derived from the M2 allosteric hexamethonium compounds W84 and naphmethonium (NAM regarding orthosteric agonists) linked with the non-selective orthosteric superagonist iperoxo. The dualsteric compounds possess relatively good selectivity for the M2 receptor regarding potency and affinity. Agonistic activation and unexpected selectivity in the intracellular G protein pathway were observed: activation of G_i_ signaling at the expense of the G_s_.

**Figure 5:**
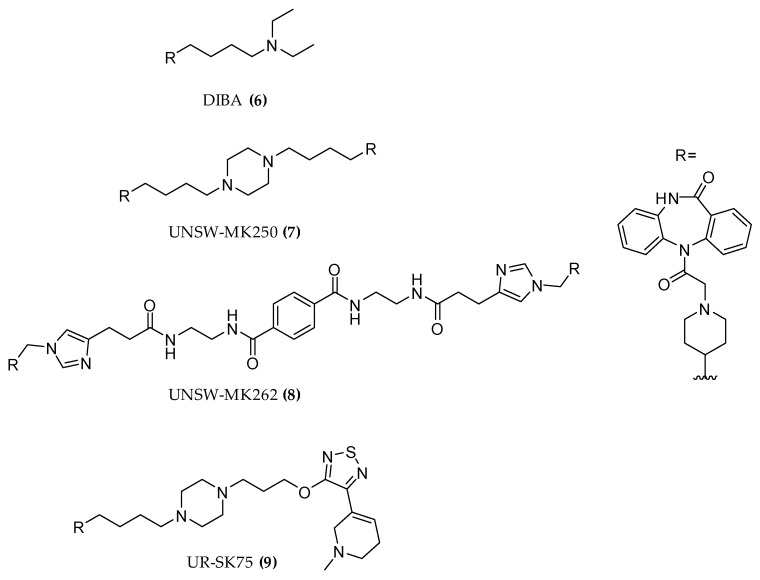
Structures of homo-hetero dimeric dibenzodiazepinone M2 derivatives.

Starting from the structure of the M2 preferring antagonist DIBA ((**6**), Figure 5), the homo-dimeric dibenzodiazepinone derivatives UNSW-MK250 ((**7**), Figure 5) and UNSW-MK262 ((**8**), Figure 5) as well as the heterodimeric UR-SK75 ((**9**), Figure 5) compounds were prepared. All molecules exhibited high M2 receptor affinities and were capable of retarding [3H]NMS dissociation, indicating an allosteric interaction [46]. Saturation binding experiments in the presence of M2 allosteric modulators and some radiolabeled dibenzodiazepinone compounds highlighted a competitive mechanism suggesting a dualsteric binding mode [47,48]. 

**Figure 6:**
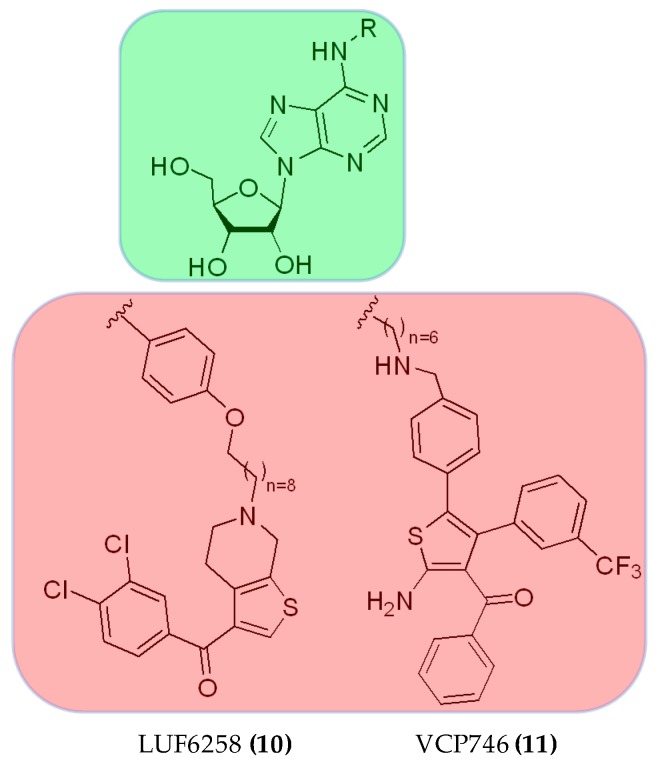
Structures of bitopic ligands **10**, **11** at A1 adenosine receptor. Green: orthosteric building block, red: allosteric building block.

Extending the approach to other GPCRs includes the adenosine A1 receptor with the dualsteric ligands LUF6258, VCP746 and VCP171 ((**10**, **11**), Figure 6; VCP171, structure not disclosed). An orthosteric adenosine agonist was connected through an alkyl chain linker to a PAM of the PD81,723 type. The resulting bitopic ligands were evaluated pharmacologically in radioligand displacement studies suggesting a dualsteric receptor interaction of LUF6258 [49]. VCP746 was subsequently developed by Valant et al. [50] by inclusion of the PAM (2-amino-4-(2-(trifluoromethyl)phenyl)thiophen-3-yl)(phenyl)-methanone) in the dualsteric structure. The bitopic ligand **11** was found to induce significant biased cellular signaling in favor of cytoprotection and to the detriment of bradycardia side effects.

**Figure 7:**
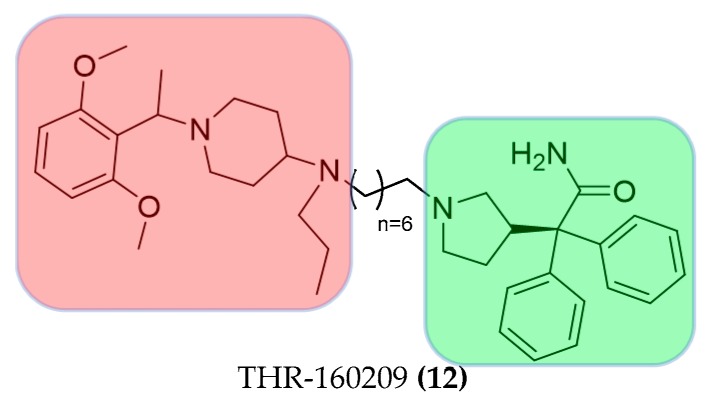
Structures of bitopic hybrids. 12 M2 acetylcholine receptor antagonist. Green: orthosteric building block, red: allosteric building block.

The compound THR-160209 ((**12**), Figure 7) was obtained by Steinfeld et al. [51] by connection of an orthosteric 3-benzylhydrylpyrrolinyl building block to an allosteric 4-aminobenzylpiperidine motif using a heptane chain. A significant increase in affinity was noted for the bitopic ligand (**12**), exhibiting a certain preference for the M2 AChR compared with its orthosteric or allosteric fragments [25,31].

**Figure 8:**
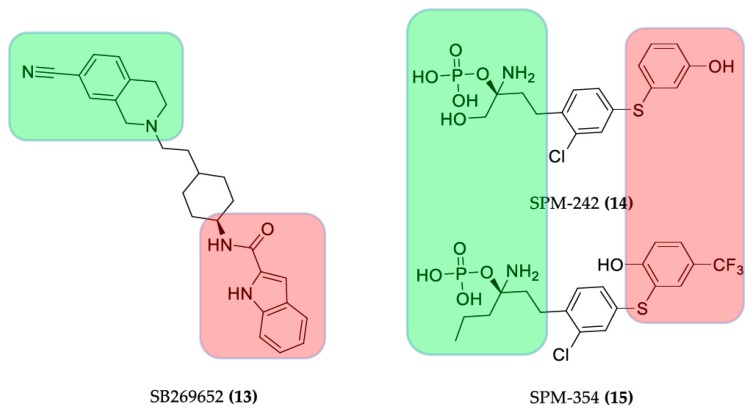
Structures of bitopic hybrids. **13** D3 dopaminergic ligand SB269652; **14**, **15** bitopic S1P3 antagonist. Orthosteric building block in green allosteric building block in red.

The dopamine D2-like receptor is object of research as drug target for neuropsychiatric disorders. The bitopic mode of action with D2 and D3 receptors was demonstrated for the dopaminergic ligand SB269652 ((**13**), Figure 8). Only a few years after its publication by Stemp et al. [52,53], SB269652 was described as an atypical bitopic ligand. It behaves like a competitive antagonist with receptor monomers and allosterically across receptor dimers by changing the ability of ligands to bind the orthosteric binding pocket on the other protomer of the dimer [54,55].

A new and potent bitopic sphingosine-1-phosphate subtype 3 receptor (S1P3) antagonist, SPM-354 ((**15**), Figure 8), with in vivo activity was a useful tool to define the spatial and functional properties of S1P3 in regulating cardiac conduction. SPM-354 can compete with both the allosteric S1P3 selective agonist CYM-5541 as well as the orthosteric agonist S1P, showing bitopic properties. SPM-354, a 2-propyl derivative of the previously described SPM242, was chosen for in vivo studies ((**14**) Figure 8) because of its improved potency and in vivo efficacy [56,57].

All these examples illustrate that this principle is applicable across the GPCR landscape. Additionally, thanks to the progress in understanding dualsteric ligands, several ligands which were thought to be allosteric modulators were re-classified later as dualsteric ligands (e.g., 77-LH-28-1 and AC-42 at M1 receptor described below see par. 4.1.4). Hence, one can be optimistic that in the future, more dualsteric ligands at different GPCRs will be discovered and designed using rational structure-based drug discovery.

#### 3.2.3. Hybrid Strategies Design

Both orthosteric and allosteric fragments and the linker play an important role in the interaction with the target protein, thus an optimization process considering these moieties is indispensable. Both pharmacophores have to be enhanced separately in a kind of fragment-based design approach, investigating the chemical environment of the two binding pockets. However, one should remember that the binding of one pharmacophore is influencing the binding of the other moiety. For example, it is possible that upon binding of the ligand the receptor goes through conformational changes causing negative cooperativity for the complementary moiety. Therefore, it is difficult to predict the pharmacological outcome of such bitopic molecules. Furthermore, the linker must fit perfectly, allowing the allosteric and orthosteric fragments to reach their target pockets. Length, flexibility, connection position, and chemical characteristics of the linker play a fundamental role in the bitopic interaction.

Bitopic ligands range from the first combination of agonist-NAM (e.g., iperoxo-6-phth) [58] to agonist-PAM (e.g., iperoxo-BQCAd) [49,50,59,60] and antagonist-NAM (e.g., atr-6-naph) [61] for the M2 and M1 muscarinic receptors. All possible combinations easily widen the spectrum of opportunities.

Alternative approaches may also include the use of bitopic ligands to study the low-affinity and transient binding sites which are defined as metastable sites [62]. Several research groups have hypothesized the existence of such a binding site at the entrance of the receptor to which ligands bind transiently [63,64,65,66,67,68]. If an energetically favorable conformation change of the complex ligand-receptor occurs, the ligand continues its path down to the orthosteric site whereas otherwise, its route is prevented through dissociation. This metastable site acts as a selectivity filter: access is denied to molecules that do not have the required physico-chemical characteristics for the binding site. Metastable binding sites offer new receptor sections that can be explored, opening new strategic opportunities in the field of bitopic ligands. 

Since several GPCRs are organized in dimeric or oligomeric complexes, respectively, bivalent ligands intended to target an allosteric and orthosteric binding sites or even two orthosteric sites of two different protomers in a receptor dimer. This will constitute a new design prospective. Rather than promoting a ligand-induced dimerization, the bivalent ligands tend to stabilize the pre-existing dimers. Unique pharmacological properties including receptor selectivity could be achieved as bivalent ligands are able to bridge different receptor units. Heterodimers can be highly selective compounds targeting only those tissues in which both receptors are co-expressed. Apart from the opioid receptor field, the potential of these ligands has been poorly explored up to now [69].

The bitopic approach used for the first time in 2006 by De Amici et al. [70] on the M2 muscarinic receptor was recently extended in the research field of AD on M1 receptor.

## 4. Muscarinic Receptor

The muscarinic receptors are involved in diverse functions throughout the body with particular focus on the bladder, gastrointestinal tract, eye, heart, brain, and salivary glands [71]. Physiologically, these functions are activated and deactivated selectively thanks to the specific tissue distribution of receptors and the existence of five different receptor subtypes (M1–M5). The M1-type muscarinic AChRs, predominantly expressed in the hippocampus and cerebral cortex, play a central role in cognitive processing, memory, and learning. Since structure and density of these AChRs remain almost unchanged in the disease state, the M1 receptor is considered a very promising target to increase the cholinergic tone [72]. 

Highly subtype selective drugs are needed in order to avoid side effects originating from unwanted receptor activation or inhibition [38]. A number of M1 muscarinic agonists was developed to treat AD. These are promising agents as they display significant neurotrophic effect, decrease β-amyloid plaque deposition, and improve oxidative stress-induced damage. Several compounds have shown an efficacy on the M1 as a therapeutic target for the AD as they not only enhance the cholinergic functions but can also shift the APP (amyloid precursor protein) processing towards the nonamyloidogenic pathway through activation of protein kinase C, the metalloproteinase domain 17 (ADAM17), and a disintegrin [3]. Activation of M1 muscarinic receptors attenuates tau hyperphosphorylation via glycogen synthase kinase-3b (GSK-3b) inhibition, reduces β amyloid peptide levels, and increases ERK activation and potentiation of NMDA receptors [38,73,74].

**Figure 9:**
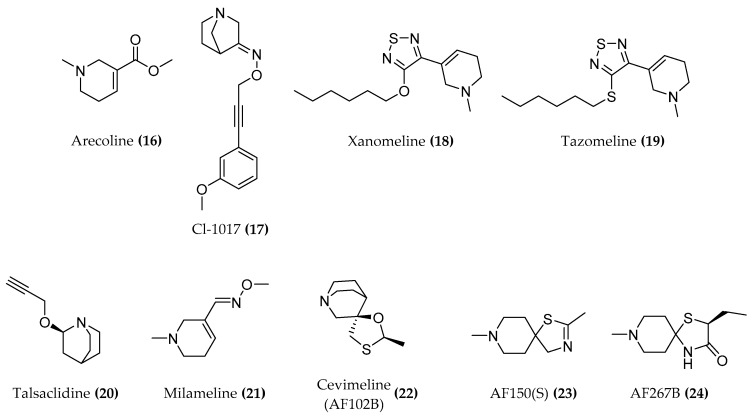
Structures of moderately active and selective orthosteric M1 agonists.

Considerable efforts were made to obtain agonists for M1 but their off-target effects preclude the therapeutic use. Various agonists for M1 muscarinic receptor including arecoline ((**16**), Figure 9) CI-1017 ((**17**), Figure 9), xanomeline ((**18**), Figure 9), tazomeline ((**19**), Figure 9), talsaclidine ((**20**), Figure 9) and milameline ((**21**), Figure 9) do not show sufficient selectivity for the M1 subtype. The most prominent candidates in the past few years were the M1 muscarinic agonists AF102B ((**22**), Figure 9) (cevimeline; approved in the USA and Japan for Sjogren’s Syndrome and having excellent pharmacokinetic properties), AF150(S) ((**23**), Figure 9) and AF267B ((**24**), Figure 9) which additionally show a significant neurotrophic effect, inhibit Aβ and oxidative stress induced cell death as well as apoptosis in PC12 cells transfected with the M1 muscarinic receptor [5,75]. 

**Figure 10:**
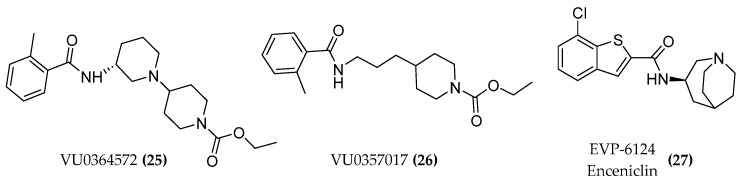
Structures of M1 lead candidates.

Furthermore, the M1 selective agonist lead candidates VU0364572 ((**25**), Figure 10) and VU0357017 ((**26**), Figure 10) were tested on cell lines as well as animal models and exhibited encouraging as well as effective properties on various parameters. The compound VU0357017 not only showed excellent M1 subtype selectivity but also a good blood brain barrier (BBB) penetration and in vivo efficacy [38]. EVP-6124 (enceniclin) ((**27**), Figure 10) was the last cholinergic receptor agonist which entered phase 3 trials but it was withdrawn due to severe gastrointestinal adverse effects [3].

**Figure 11:**
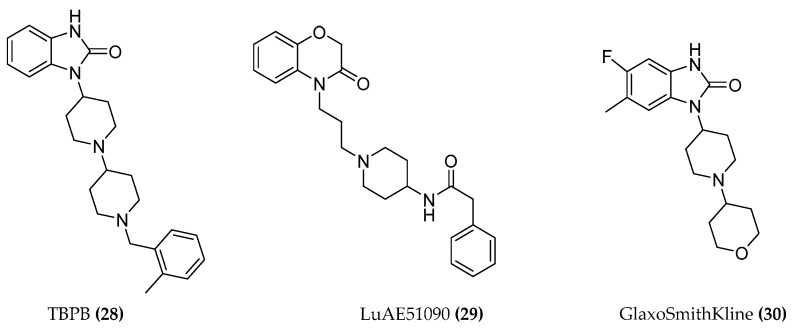
Structures of M1 allosteric agonists.

Some highly M1-selective allosteric agonists must also be mentioned here which do not require an orthoster bound to the receptor for activation. For example, the TBPB allosteric agonist ((**28**), Figure 11) interacts with slightly different binding domains of the M1 receptor which are all adjacent to the orthosteric site. LuAE51090 ((**29**), Figure 11) displayed a high degree of selectivity when tested in a broad panel of GPCRs, ion channels, transporters, and enzymes, and showed an acceptable pharmacokinetic profile and BBB penetration properties in vivo [76]. Compound **30** (Figure 11) was identified by high throughput screening (HTS) and virtual screening at GlaxoSmithKline, unraveling a molecule with improved binding and pharmacokinetic parameters. For **30**, good M1 agonist potency, intrinsic activity, and subtype selectivity for M1 are reported [77,78].

Both the comparison of the allosteric agonists among each other and even with M1-PAMs reveals structural similarities. They all consist of an heterobicycle system with at least one nitrogen atom and a carbonyl group close to the nitrogen. Furthermore, the heterocycle is connected to piperidine rings which are linked to another cyclic moiety. 

### 4.1. Design of Hybrid Compounds to Investigate the Muscarinic Receptor

The allosterically less conserved site of the mAChR which is located in the extracellular vestibule (ECV) and separated from the orthosteric pocket by a tyrosine lid [79] lends itself well as binding site for new dualsteric ligands. The close proximity of the orthosteric and the allosteric subdomains, a typical feature of the Class A GPCRs, allows the dualsteric compounds to simultaneously interact with the two binding sites.

**Figure 12:**
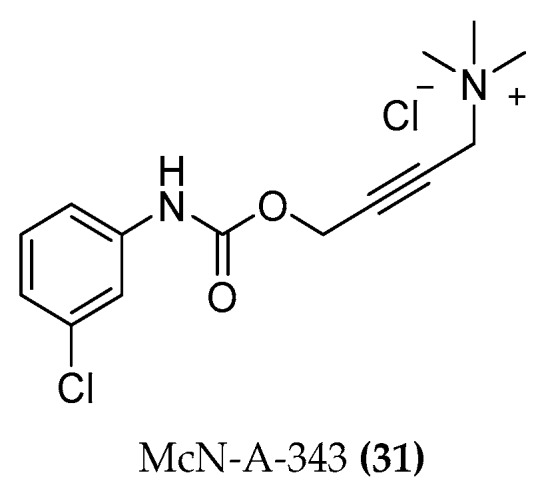
Structures of McN-A-343.

The chemical space between the orthosteric and allosteric portions of the M2 receptor was investigated thanks to the pharmacological analysis of Iper-8-naph ((**5b-C8**), Figure 4) [80,81], McN-A-343 ((**31**), Figure 12), and its fragments and analogues [82].

The long-known partial agonist McN-A-343 interacts with the allosteric EL2 vestibule through its 3-chlorphenylcarbamate. The binding to the orthosteric sites is guaranteed by the tetramethylammonium cation. The two parts are linked via a rigid butynyl chain. However, this molecule is probably too small to entirely occupy the allosteric pocket and thereby achieves no subtype selectivity. It binds to both M2 and M4 receptors [31].

Iper-8-naph is a partial agonist at the M2 receptor. The partial agonism of most of the dualsteric agonists depends on both the dualsteric binding mode itself and on the dynamic equilibrium of multiple inactive and active states, i.e., the ligand binding ensemble. In the “dualsteric binding mode”, iperoxo is bound to the orthosteric pocket while the allosteric naphthalimide moiety protrudes towards the extracellular part of the receptor. This pose is responsible for a functionally selective activation of the G proteins and cellular signaling [81,83]. In the alternative pose, which is a “purely allosteric binding mode”, the entire bitopic ligand (both the orthosteric and the allosteric moieties) is in the allosteric vestibule. In this case, the inactive receptor conformation is stabilized. It would be difficult to pharmacologically distinguish the “flip-flop” mechanism from a ligand with a purely bitopic binding mode. In case of the iperoxo-dualsteric derivatives at the M2R, the bitopic mode of interaction was validated by the following experiments: radioligand binding analysis, in wild-type and mutant receptors, and downstream signaling assays in addition to receptor docking simulations [34]. Partial agonism is the direct consequence of this dynamic balance. Measuring the partial agonism depends on the affinity of the two pharmacophores and of the dualsteric ligand itself for the respective binding sites [30].

#### 4.1.1. BQCAd Hybrid Derivatives

**Figure 13:**
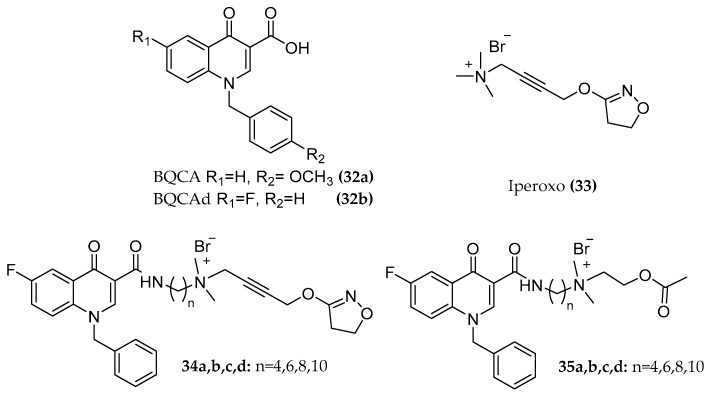
Structures of BQCAd/Iperoxo-ACh hybrids.

The same approach was applied for exploring the M1 receptor binding pockets. Among several selective M1 allosteric modulators known, the promising group of *N*-benzyl quinolone carboxylic acids (BQCAs) was selected as positive allosteric moieties of these dualsteric M1 hybrids [59,60]. The BQCA allosteric modulator ((**32a**), Figure 13) was first described by the Merck laboratory. The compound is a positive allosteric modulator, an allosteric agonist, and exhibits pure subtype selectivity for the M1 receptor: no agonism, antagonism, nor potentiation activity was observed at other mAChRs up to 100 µM. The concentration of ACh which needed to activate the receptor decreased up to 129-fold in the presence of BQCA and an inflection point of 845 nM is reported in the presence of 3 nM ACh. Its allosteric modulation is mediated by the interaction with the residues Y179 and W400, suggesting the binding of BQCA to the "common" allosteric site [84,85]. As an orthosteric fragment, the non-selective superagonist iperoxo ((**33**), Figure 13) [59] and the endogenous ligand ACh were linked to the BQCA derivative (BQCAd ((**32b**), Figure 13)) [60]. The two parts were connected by alkylene chains of different lengths (C4, C6, C8, C10) ((**34a, b, c, d**), Figure 13).

The compounds showed

1. subtype selectivity: receptor activation and signaling pathway studies performed with (dynamic mass redistribution) DMR in the presence and absence of specific G_i_ and G_q_ signaling blockers indicate a preferentially Gq mediated signal of the hybrids.
2. partial agonism which may provide controlled activation without overdriving the M1 signaling and with possible improved side effect profile over the control of cognition [86].
3. potency and efficacy dependency on the length of the chain and on the substitution pattern of the allosteric fragment. Elongating the chain from C4 to C6 switches the receptor fractional occupancy from the inactive, purely allosteric pose to the active binding pose which defines the efficacy of the dualsteric ligands [87].

**Figure 14:**
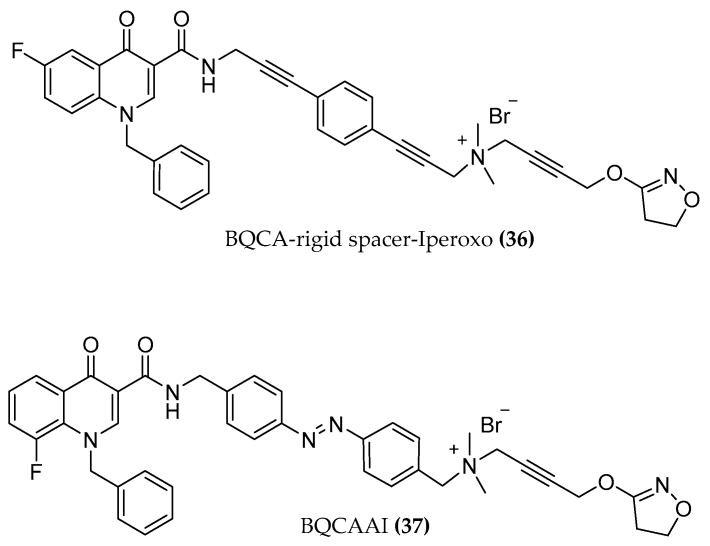
Structures of BQCAd/Iperoxo hybrids connected by a rigid linker and a photo-switchable moiety.

The interaction of these compounds with the M1 muscarinic receptor was further investigated by Fluorescence Resonance Energy Transfer (FRET) measurements in a living cell system for a closer understanding of the receptor movement. Here, some other ligands were added to the hybrid series including longer chained (C8, C10) compounds and ACh hybrids ((**35a,b,c,d**), Figure 13) [60]. The optimal linker length for the activation of the receptor was confirmed to be a C6 chain, e.g., in the case of iperoxo-C6-BQCA with a maximal detected signal of about 24% compared to 100 µM of the iperoxo reference. Surprisingly, a new receptor conformation was observed: a FRET signal with opposite direction to the typical agonist signal occurred for the longer chain hybrids. How this unknown receptor activation process at a molecular level is connected with the bitopic compound interaction mode, the different G-protein activation, β-arrestin recruitment, or further downstream signaling must be investigated to completely understand this unexpected M1 receptor arrangement. The distinct receptor movement that occurs upon binding of dualster ligands may help to illuminate the interplay between G proteins and β-arrestins preferentially coupling to different receptor conformations [88]. Particularly within the M1 pharmacology, functionally selective properties can be exploited to characterize which signaling outcomes would address neuropsychiatric illnesses [89]. Of note, replacing the flexible alkyl chain with a rigid alkynylbenzene linker ((**36**), Figure 14) leads to a delayed FRET signal, indicating slower receptor kinetics. The compound with a flexible chain of corresponding length afforded a sharper FRET signal, indicating the responsibility of the rigidification in the kinetic modification [60,90]. 

**Figure 15:**
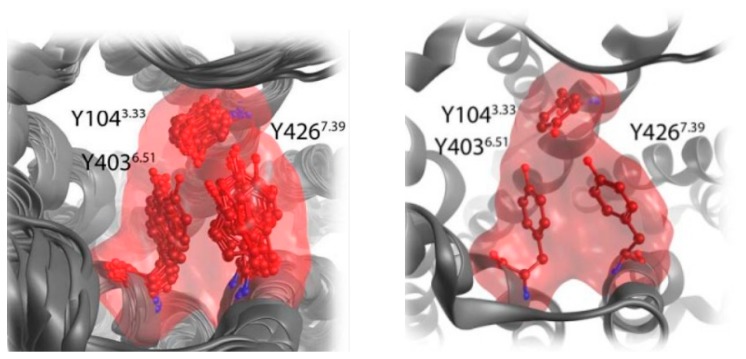
Left: top view snapshots from a molecular dynamics simulation of the iperoxo-bound crystal structure unveil the conformational flexibility of the tyrosine lid, especially Tyr-426^7.39^. Right: based on the sampling of side chain conformations, the tyrosine lid was remodeled for binding mode investigations on dualsteric agonist. Reproduced with permission from [83].

Probably, a cation-π interaction between the three tyrosines Y104^3.33^, Y403^6.51^ and Y426^7.39^ of the so-called tyrosine lid with the rigid linker contributes to the delayed FRET signal. (Remodeling of the tyrosine lid. in Figure 15). 

A further step in the study of M1 activation processes was performed with the synthesis and pharmacological investigation of the first new photo-switchable dualsteric ligand. Agnetta et al. [90] designed a pharmacological tool in which the photochromic azobenzene moiety was inserted as a spacer in the hybrid compound. Regarding the time course of M1 activation, compound **37** was found to be as slow as with the rigid compound, underlining the fundamental role of the chain structure of the dualsteric hybrids in the M1 receptor kinetic. More interestingly, with the BQCAAI ((**37**), Figure 14) compound, the drastic influence of the iperoxo-BQCA hybrid geometry on the activity of the compound was demonstrated. A simple light-induced isomerization from the *cis* to the less stable *trans* BQCAAI isomer turns the antagonist compound into an agonist. 

#### 4.1.2. LY593093

**Figure 16:**
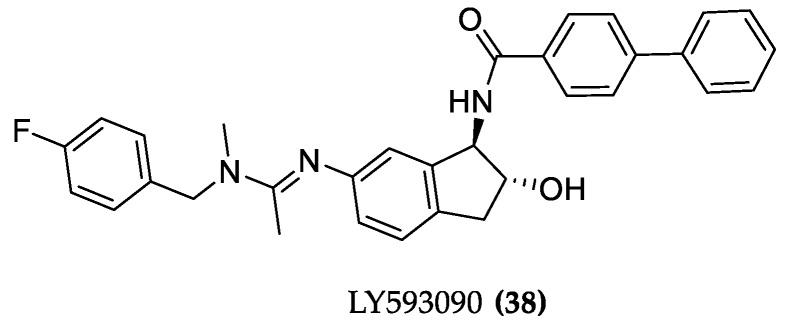
Structure of LY593093.

Another allo/ortho partial agonist hybrid was designed at Eli Lilly: LY593093 ((**38**), Figure 16). The molecule displaced the [^3^H] NMS antagonist from M1 muscarinic receptor (pK_i_ of 6.21) without any effect on [^3^H] NMS binding at the M3-M5AChR and stimulating β-arrestin recruitment. Additionally, LY593093 had no modulatory role in potentiating ACh functional activity and the binding was not affected by an increasing concentration of the PAM BQCA, indicating that the interaction between LY593093 and M1AChR is different from the interaction of the receptor with the endogenous ligand ACh. High subtype selectivity is achieved through multiple points of binding pocket interactions which was shown in the docking model [86]. Binding was also observed for the M2 receptor, but in terms of functional activity the Eli Lilly compound is 5 and 120 times more potent on M1 AChR than on other muscarinic receptor subtypes and shows an E_max_ at least 2-fold higher [91]. Progression through clinical trials was however interrupted due to its modest brain penetration.

#### 4.1.3. AC-42 and AC-260584

**Figure 17:**
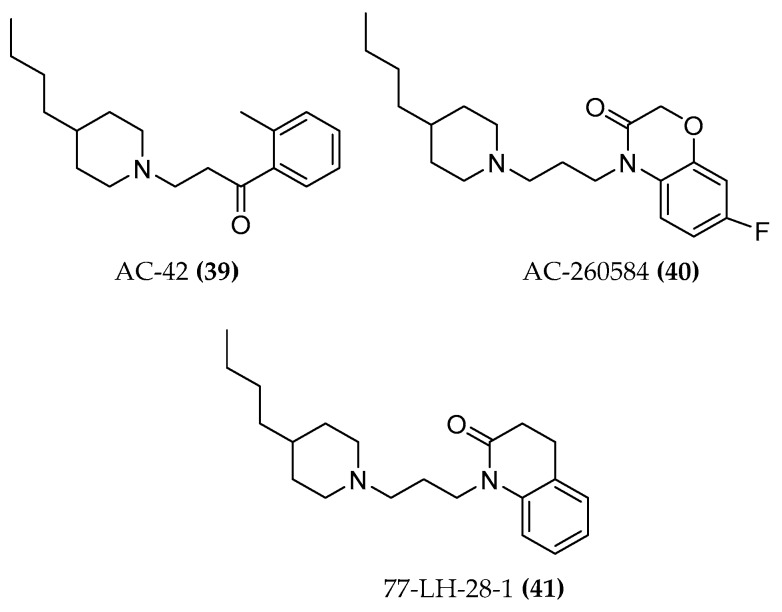
Structures of AC-42, AC-260584 and 77-LH-28-1.

The allosteric and orthosteric mode of action was also demonstrated by means of a combination of functional and binding assays for the AC-42 compound ((**39**), Figure 17). The compound slows down the dissociation of [^3^H]NMS from the M1 muscarinic receptor, indicating its allosteric capabilities; moreover, in equilibrium binding studies, AC-42 almost completely inhibits the binding of the [^3^H]NMS orthosteric probe and, in the functional test IP1, the antagonist atropine decreases the potency of the ligand in a concentration-dependent manner. Later findings indicated an additional orthosteric binding pose of the ligand [2,92,93]. AC-42, unlike oxo-M, arecoline, and pilocarpine, is unable to promote M1-G_αi1/2_ protein coupling, proving the ability of bitopic ligands to activate different down streaming effectors [94]. AC-260584 ((**40**), Figure 17), the orally bioavailable and structurally related compound with greater potency and efficacy than AC-42, was initially defined as an allosteric agonist. Mutation of amino acids in the TM3 of the M1 receptor (ACh binding site) affects the binding of the compound and hence supports the hypothesis of the bitopic behavior [95].

#### 4.1.4. 77-LH-28-1

77-LH-28-1 ((**41**), Figure 17), a close structural analogue of AC-42, was initially described as an allosteric agonist. However, it might have a bitopic binding mode because the antagonist scopolamine is able to shift its dose-response curve rightward indicating an orthosteric binding mode. Further evidence supporting this theory is the competition at the NMS-occupied receptor with a prototypical muscarinic negative allosteric bis-quaternary phthalimidopropyl modulator (heptane- 1,7-bis(dimethyl-3′-phthalimidopropylammonium bromide, abbreviated as C7/3-phth) [92]. 

77-LH-28-1 was, however, used as an allosteric fragment of the bitopic hybrids synthesized by Piergentili et al. [89] The compounds containing xanomeline as orthosteric fragment were pharmacologically investigated as described below (See par. 5.1.4).

## 5. Acetylcholinesterase (AChE)

**Figure 18:**
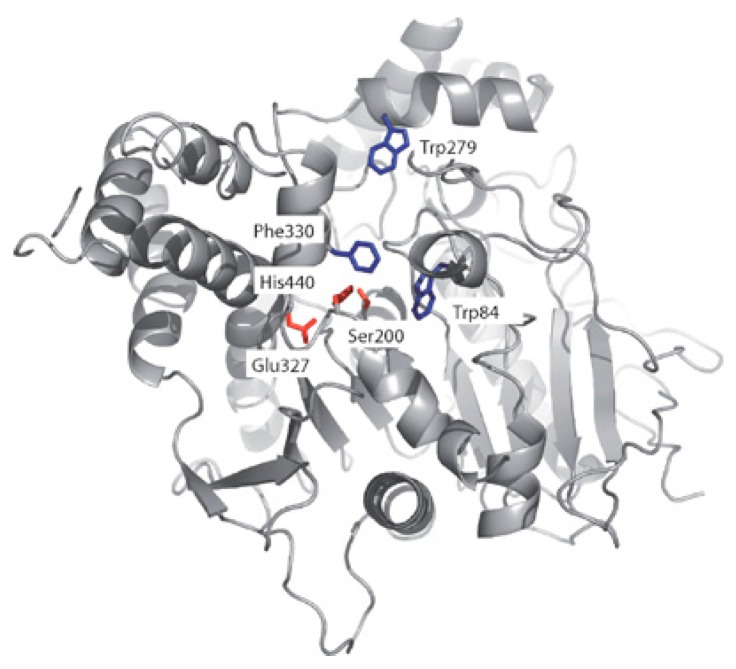
3D structure of native *torpedo californica* AChE. The catalytic triad in red, Trp84 in the CAS, Trp279 at the PAS, and the bottleneck residue Phe330 in blue. Reproduced with permission from [99].

The decreasing cholinergic tone is one of the physiological reasons of the debilitating AD. To switch off a cholinergic signal, ACh is catabolized by the acetylcholinesterase (AChE) which hydrolyzes the ester of the neurotransmitter to acetic acid and choline. The enzyme also appears to be involved in the aggregation of Aβ by a peripheral anionic site (PAS) in addition to its classical enzymatic hydrolysis of ACh [96,97]. AChE inhibitors compensate the cholinergic tone in the synaptic transmission and thus, because of an augmenting ACh concentration, usually relieve the symptoms and can decrease the rate of cognitive decline for some months. However, they are not able to arrest disease progression and inexorable neurodegeneration [27]. Already in 1991, Sussman et al. described the enzyme as a narrow cavity that drives the substrate through a peripheral anionic site (PAS) to the catalytic site (CAS), where the catalytic triad Glu-His-Ser is located [98]. (Figure 18).

**Figure 19:**
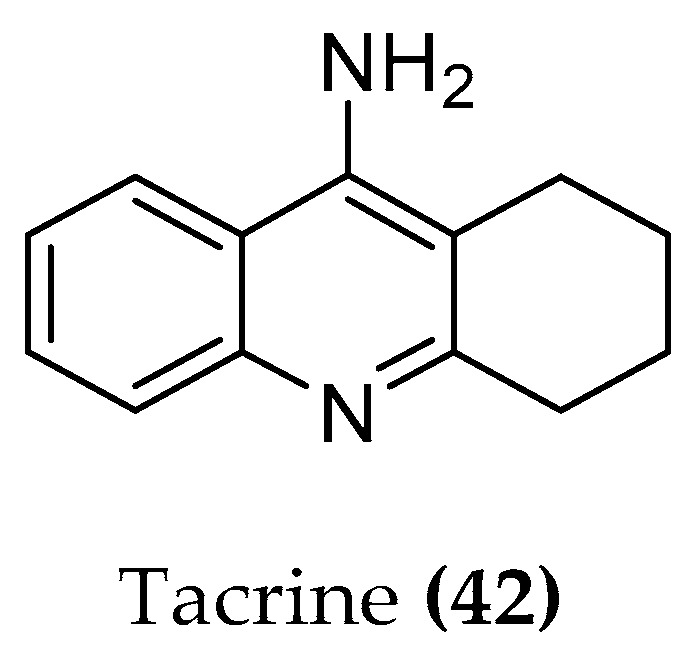
Structure of tacrine.

The knowledge of the active site and of the exact function of each amino acid of the catalytic triad allowed the discovery of enzymatic inhibitors. The AChE inhibitors (AChEIs) donepezil, galantamine, rivastigmine ((**1**–**3**), Figure 1), and tacrine ((**42**), Figure 19) were successfully developed and approved by international authorities. Later, tacrine was withdrawn from the market due to a significant hepatic toxicity.

**Figure 20:**
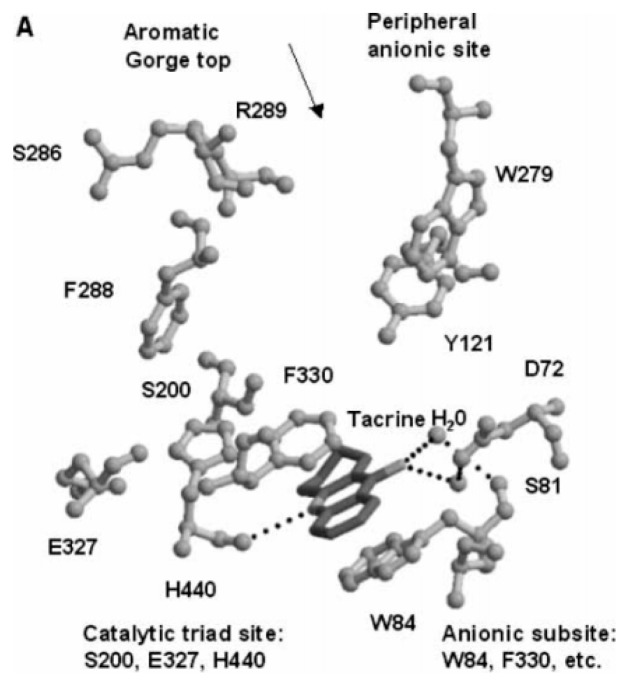
Overlay of the trigonal crystal structure of tacrine (black) and Tc AChE (gray) complex: showing the catalytic anionic site, aromatic gorge, and peripheral anionic site. Reproduced with permission from [100].

These compounds inhibit the enzyme differently: several ligands, e.g., galantamine, covalently bind to the serine residue and form a bond that is stable for about 15–20 min; during this time, the enzyme is inhibited. Tacrine blocks the passage of the neurotransmitter due to a strong π-stacking interaction with Trp84 (Figure 20) [100]. 

Rivastigmine is a pseudo-irreversible arylcarbamate inhibitor of both AChE and BChE. Clinical studies comparing the effect of the two approved drugs donepezil (only AChE inhibition) and rivastigmine (AChE and BChE inhibition) revealed an additional benefit in daily living, global function, and behavior for rivastigmine in patients with wild-type BuChE allele due to the dual inhibition of AChE and BChE [101,102,103]. Anyway, the use of AChEIs for AD is considered a suboptimal therapy and is still under discussion [104]. This review will not give details about all AChEIs because they were already summarized excellently in ref. [105,106,107,108]. 

### 5.1. Design of Hybrid Compounds Inhibiting the Cholinesterase

#### 5.1.1. Tacrine Hybrids

**Figure 21:**
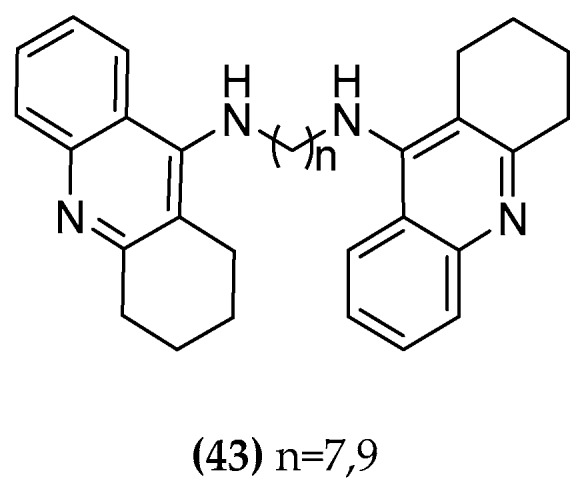
Bis tacrine homodimers.

In order to investigate the potency of tacrine for the inhibition of AChE with simultaneously reduced hepatotoxicity, tacrine was intensively used for the development of hybrid molecules. Pang et al. found a bis-C7-tacrine homodimer ((**43-C7**), Figure 21) exhibiting a 1000-fold higher AChE inhibitory potency in rats than the monomer, indicating the absence of steric interaction problems in the narrow cavity preceding the catalytic site of the enzyme. The tacrine dimers are able to establish interactions with both the catalytic and the peripheral site. A shorter tether (<5 methylene units) hampers the simultaneous binding to both interaction sites of AChE (dual-site binding). In addition, the lipophilicity of the hybrid was found to be increased compared to tacrine itself [109,110].

#### 5.1.2. Tacrine/Iperoxo hybrids

**Figure 22:**
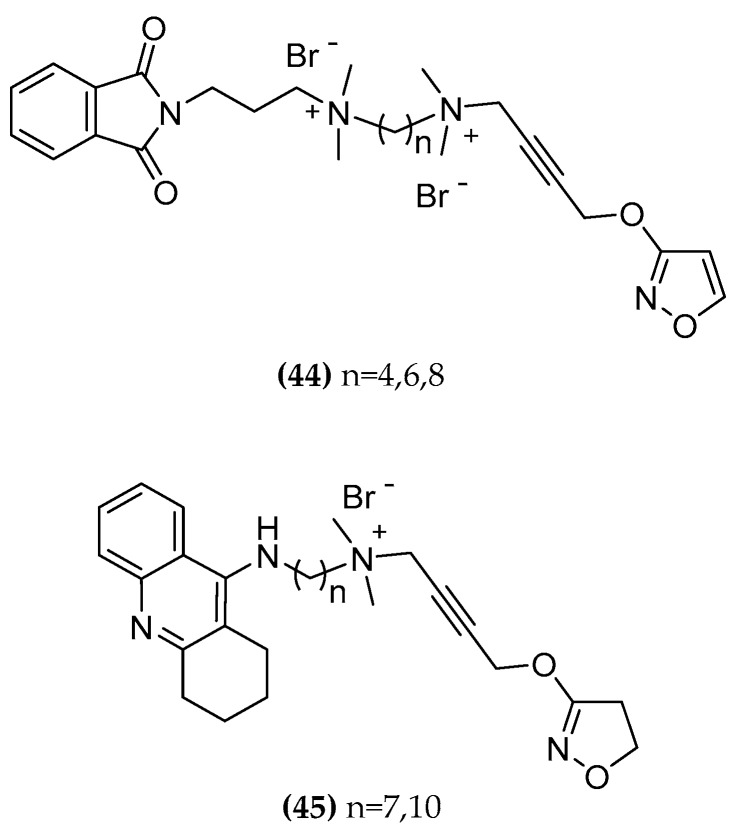
AChE inhibitor/ mAchR agonist compounds.

Some bitopic ligands combining the M2 allosteric structure of the allosteric modulator W84 and naphmethonium and the non-selective superagonist iperoxo, rationally developed as M2 subtype-selective ligands ((**5a,b**-**C7,8,9,10**), Figure 4), were tested for the inhibitory activity of the AChE and found to have modest activity. The potency is influenced by the length of the linker chain connecting the orthosteric and allosteric sites. The longest chains result in a higher activity: the decamethylene chain seems to span the AChE binding gorge, allowing the quaternary ammonium head of iperoxo to be localized in the PAS region. Compounds containing the naphthalimide portion ((**5b-C7,8,9,10**), Figure 4) show stronger inhibition than those containing the phthalimide part ((**5a-C7,8,9,10**), Figure 4). When replacing the iperoxo moiety with an isoxazole ring, almost no AChE/BuChE inhibition was observed ((**44-C4,6,8**), Figure 22; Table 1) [80].

Starting from this finding, new hybrids with multitarget potential ((**45**), Figure 22) were synthesized consisting of a tacrine and an iperoxo moiety, aiming at higher inhibitory activity for the AChE and BChE [111]. The idea was to combine the iperoxo affinity for the M1 and M2 receptor with the AChE inhibitory activity. Tacrine can serve as both an allosteric modulator for M receptor and as inhibitor of AChE [112,113,114]. In particular, Tac-10-iper ((**45-C10**), Figure 22) was an extremely potent inhibitor with an IC_50_ value of 9.81 (AChE from electric eel; tacrine IC_50_ 7.60) and 8.75 (BChE from equine serum; tacrine IC_50_ 8.57) which is even better than tacrine itself. Moreover, it shows less cytotoxicity.

**Figure 23:**
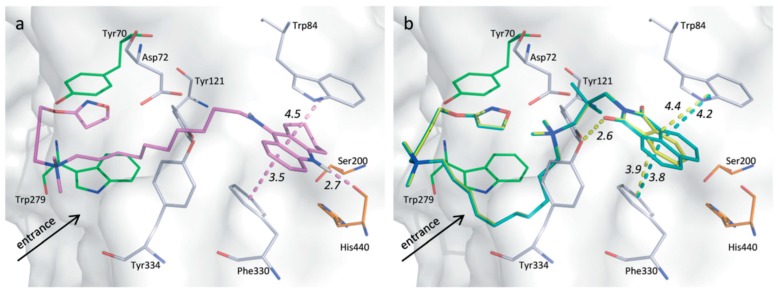
(**a**) Docking pose of **45-C10** (pink), with the interaction distances shown in the CAS (orange) and in the PAS (green). (**b**) Docking representation of **5b-C10** (blue) and **5a-C10** with their interaction distances shown in yellow. Reproduced with permission from [110].

Docking studies show a similar binding mode of compounds **5a-C10** and **5b-C10** to Tac-10-iper ((**45-C10**) in the CAS and PAS regions of AChE (Figure 23). The remarkably lower affinity of **45-C10** is probably due to the additional quaternary ammonium group of the Phtal- and Naph-compounds which causes a much higher desolvation penalty in comparison to the uncharged alkylene chain.

A dual mechanism of action which appears to be the right innovation to fight the AD pathology may also be possible due to the high affinity for M1 and M2 receptors. Furthermore, functional studies (investigation of Ga and PLC-b3 proteins interaction through Split-Luciferase assays) demonstrated the ability of Tac-7-Iper and Tac-10-Iper ((**45-C7**, **45-C10**, Figure 22) to activate the M1 muscarinic receptor in a nanomolar range (Volpato et al., unpublished results). Further studies are necessary for developing uncharged molecules that could cross the BBB; so far, it is promising to see that compounds with a single positive charge are associated with better activity than compounds with two charges [110,111]. 

#### 5.1.3. Tacrine/Xanomeline Hybrids

**Figure 24:**
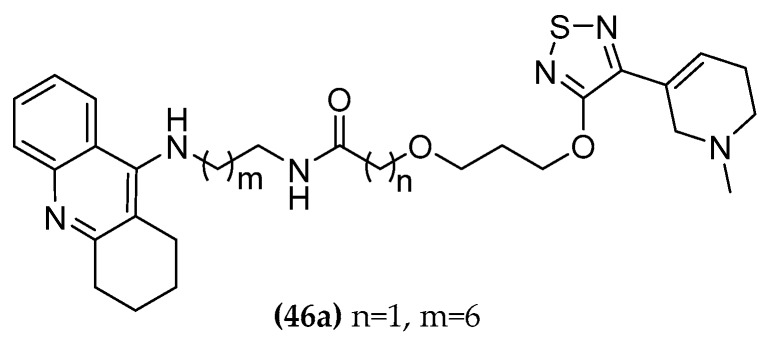
Dual-active AChE inhibitor/mAChR agonist compounds.

A similar dualsteric strategy was previously employed by the Decker group [114]. They designed and synthesized tacrine and xanomeline hybrid compounds ((**46a**), Figure 24). The rational design of these hybrids was aimed at a triple mechanism of cooperative action: activation of the M1 receptor through the xanomelinic molecular portion ((**18**), Figure 9), positive allosteric modulation regarding orthosteric agonists at the M1 receptor subtype thanks to the allosteric properties of tacrine, and increasing the amount of ACh in cholinergic synapses due to the AChE inhibiting activity [114]. Xanomeline is an M1 agonist but unfortunately also shows some affinity to the M4 receptor. Therefore, parasympathetic side effects including bradycardia, increased gut motility, and salivation occur. Binding studies on cloned human muscarinic receptors did not detect the selective binding capacity between M1 and M4. Xanomeline is well-known for its anti-dementia properties *in vivo*, and the attenuation of cognitive decline in 6 months clinical trial human patients proved its activity against AD [115]. The metabolic instability due to an extensive first-pass metabolism, the well-known cholinomimetic side effects, and its undesirable activity for the dopaminergic and 5-TH receptors (serotonin receptors) rendered the compound an insufficient clinical candidate [116,117]. Xanomeline induces a specific mode of M1 receptor activation by interacting with a site that does not fully overlap with the endogenous, orthosteric binding site. Differences in binding kinetics [118] and a wash-persistent binding, probably arising from a hydrophobic interaction of xanomeline’s O-hexyl chain with the membrane lipids surrounding the receptor [119,120], may explain its functional selectivity [121].

The xanomeline portion was connected to the tacrine with amide bridges of different length (10-17 atoms spacer). A clear increase of AChE inhibition was observed by increasing the spacer chain from pIC_50_ of 7.20 to 8.21. The hybrids inhibited the AChE with similar or higher potency compared to tacrine but did not activate the M1 receptor. Instead of a simultaneous allosteric/orthosteric binding, the ligands seem to prefer a purely allosteric binding even in the orthosterically free receptor. These findings were supported by in vivo studies in rats with a representative compound ((**46a**), Figure 24). The cognitive deficits induced by administration of scopolamine were enhanced by the purely M1 allosteric interaction of the hybrid **46a** counterbalancing its high ChE-inhibitor properties. The desired triple mechanism of action unfortunately did not take place with these compounds. This demonstrates the difficulty of predicting the intrinsic activities of the chosen component molecules and the importance of their cooperativity, which must lead to a balanced compromise.

#### 5.1.4. 77-LH-28-1/Xanomeline Hybrids

**Figure 25:**
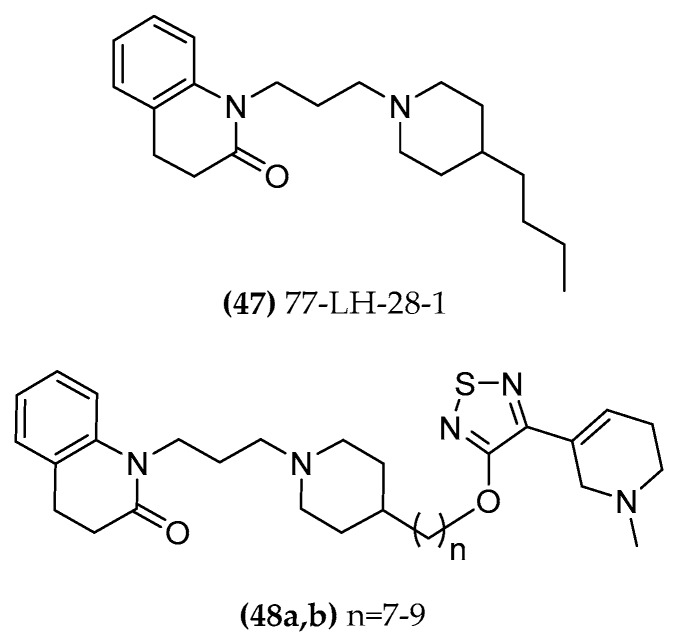
Dual-active AChE inhibitor/ mAChR agonist compounds.

As already mentioned above, compound 77-LH-28-1 ((**47**), Figure 25) displaying mixed modes of orthosteric or allosteric pharmacology depending on the experimental assay conditions [122], was incorporated into a series of allo/ortho hybrid ligands, hypothesizing a shared function of the common chain ligand structures of xanomeline and 77-LH-28-1 ((**48a,b**), Figure 25). With a merging approach, the two scaffolds were fused into the new hybrids, aiming for a better understanding of the allosteric pocket and of the bitopic binding pose of 77-LH-28-1 itself.

The pharmacological investigation of these hybrid derivatives evidenced an affinity at the M1 receptor higher than 77-LH-28-1 and lower than xanomeline. In general, hybrid compounds showed either a weak agonistic or an antagonistic activity profile (M1 and M4) in all the functional assays and the activity/selectivity was related to the length of the spacer. The highest affinity values are associated with a seven- or nine-atom length carbon chain, indicating that the two pharmacophores probably interact with two distinct sites located at a distance of 7 to 9 methylene moieties. Xano-9-77-LH-28-1 exhibited higher efficacy for the β-arrestin2 engagement in M1. In line with the reported functional properties of the aliphatic chain moiety in 77-LH-28-1, these results indicate that the linker extension affects the orthosteric binding site [89].

## 6. Nicotinic Receptor

Nicotinic receptors are transmembrane pentameric proteins that belong to the superfamily of ligand-gated ion channels, composed of α and β subunits assembled around a central hydrophilic pore that mediates the flow of the cations K^+^, Na^+^, and Ca^2+^. Different combinations of subunits generate a variety of nAChR subtypes with distinct electrophysiological properties and brain localization [123,124]. nAChRs are considered to play an important role in neuroprotection due to a concentration dependent interaction with the β-amyloid peptide. In particular, the physiological concentration of Aβ may directly stimulate the α_7_ nicotinic receptors while an increasing pathological concentration of Aβ damages the cholinergic responses mediated by α_7_ and α_4_β_2_ receptors. 

### 6.1. Design of Hybrid Compounds to Investigate the Nicotinic Receptor

#### 1-(2-Methoxybenzyl)-Piperazine-Carbazole Hybrids

**Figure 26:**
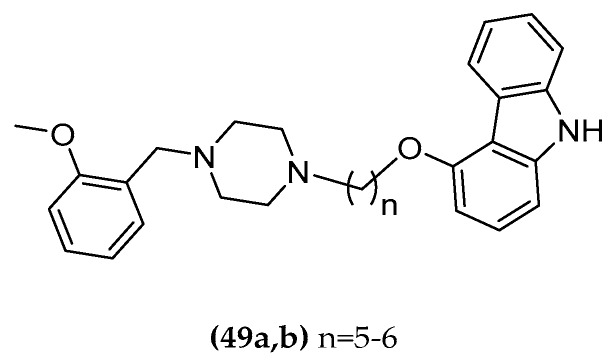
Dual-active AChE inhibitor/ nAChR agonist compounds.

The mutual interaction between Aβ and cholinergic transmission was investigated. Following a ligand-based approach, hybrid compounds addressing both systems were synthetized and subsequently pharmacologically studied. The molecules combine an α_7_ agonist and a carbazole moiety having AChE inhibition properties ((**49a,b**), Figure 26) [125]. Substituted carbazoles are able to interact with the narrow inner part of the AChE enzyme gorge and also present anti-amyloidogenic activity. This moiety was connected with the α_7_ agonistic activity of 1-(2-methoxybenzyl)-piperazine [126]. It was selected because of the structural similarity with the ethyl-(2-methoxybenzyl)-amine group recognizing the AChE catalytic site [127,128].

The resulting multitarget compounds, in particular compound ((**49b**), Figure 26), presented AChE inhibition activity in a micromolar concentration range (compound with six methylene unit spacer IC_50_ = 0.773 µM). AChE inhibition was strongly dependent on the linker elongation. In particular, compounds with a shorter chain in the series had lower IC_50_ values. In radioligand binding assays, the compounds exhibited a micromolar affinity profile with K_i_ values ranging from 15 to 120 µM in the displacement of [^3^H]-epibatidine and [^125^I]-α-bungarotoxin from α_4_β_2_ and α_7_ receptor subtypes in rat cortex, without discrimination in the receptor subtype. The action towards nAChRs was tested by using two-electro voltage clamps. Unfortunately, the compounds only reached 15–22% of the response (reference 100 µM Ach) at α_4_β_2_ receptors. No response was observed at the α_7_ receptor, possibly due to its lower sensibility to ACh or its faster desensitization. Interestingly, their inhibition ability at 10 µM of Aβ fibril formation, proved with a ThT-based fluorimetric assay, is similar to the anti-aggregating and blocking fibril formation activity of curcumin [129] (28-36% compared to 34.4% of curcumin). Synergistically, a weak perturbation of one of the systems involved in the cholinergic neurotransmission (AChE, nAChRs) and amyloid aggregation could be sufficient to modify the entire scenario. Therefore, the compounds are a suitable starting point for further optimization.

## 7. Looking to the Future for AD Hybrids Tools

The hybrid compounds targeting different biological proteins and dualsteric ligands addressing distinct parts of the same target or a combination of both strategies provide pharmacological tools that could expand horizons in medicinal chemistry. All the hybrid examples reported provide additional useful and important information regarding their target, showing that the development of bivalent compounds is not only possible but also promising in offering new pharmacological instrumentation. Poor drugability is the direct consequence of the relatively high molecular weight of bitopic ligands which prevents their progression in drug development. The bivalent compounds can provide new useful information regarding protein-ligand interactions anyway, understanding the intricate relationship between receptor activation, conformational dynamics, signaling profile in the cholinergic system, and the connection between different hallmarks of AD. Advantages such as better selectivity, signaling bias, or reduced toxicity of some hybrids can be further exploited through a subsequent fragment based process trying to reduce physicochemical issues but keeping the precious improvement obtained. The simultaneous attack on several fronts seems to provide insights that may be the key to opening new glimpses in the cholinergic field of the AD.

## Figures and Tables

**Table 1:** Cholinesterase activity and cytotoxicity of the M2 bitopic compounds and tacrine hybrids. BChE from equine serum, AChE from electric eel. [110].

Compound	BChE pIC_50 _[M]	AChE pIC_50 _[M]	Cytotoxicity HEP G2 IC_50_ [μM]
Tacrine (**42**)	8.57	7.60	111 (±2.28)
Phth-7-iper (**5a-C7**)	5.19	4.83	n.d.^ 1^
Phth-8-iper (**5a-C8**)	5.13	4.89	n.d.^ 1^
Phth-9-iper (**5a-C9**)	4.88	4.80	n.d.^ 1^
Phth-10-iper (**5a-C10**)	5.37	5.26	n.d.^ 1^
Naph-7-iper (**5b-C7**)	6.69	6.04	n.d.^ 1^
Naph-8-iper (**5b-C8**)	6.46	6.10	n.d.^ 1^
Naph-9-iper (**5b-C9**)	6.49	6.44	n.d.^ 1^
Naph-10-iper (**5b-C10**)	6.99	6.50	n.d.^ 1^
Tac-7-Tac (**43-C7**)	9.14	10.48	<1.38 (±0.08)
Tac-10-Tac (**43-C10**)	9.34	9.00	<1.25 (±0.00)
Phth-4-isoxo (**44-C4**)	4.07	19.12% inhib. at 100 μM	n.d.^ 1^
Phth-6-isoxo (**44-C6**)	4.41	32.91% inhib. at 100 μM	n.d.^ 1^
Phtal-8-isoxo (**44-C8**)	5.10	4.17	n.d.^ 1^
Tac-7-iper (**45-C7**)	8.29	8.76	>160 (±0.00)
Tac-10-iper (**45-C10**)	8.75	9.81	32.2 (±0.41)

^1^ n.d.: not determined.
